# Prediction of Solute Segregation at Metal/Oxide Interfaces Using Machine Learning Approaches

**DOI:** 10.3390/molecules30163344

**Published:** 2025-08-11

**Authors:** Yizhou Lu, Blas Pedro Uberuaga, Samrat Choudhury

**Affiliations:** 1 Department of Mechanical Engineering, University of Mississippi, University, MS 38677, USA; ylu3@go.olemiss.edu; 2 Materials Science and Technology Division, Los Alamos National Laboratory, Los Alamos, NM 87545, USA; blas@lanl.gov

**Keywords:** metal/oxide interfaces, solute segregation behavior, density functional theory, machine learning

## Abstract

The atomic structure and chemistry at metal/oxide interfaces play a crucial role in determining their properties. However, studying semi-coherent metal/oxide interfaces that include misfit dislocations through density functional theory (DFT) is often computationally expensive due to the large number of atoms involved, ranging from hundreds to thousands. In this study, we explore solute segregation behavior at the Fe/Y_2_O_3_ interface—an important model interface for cladding applications in nuclear fission reactors—by combining DFT calculations with a machine learning (ML) approach. ML models are trained using DFT-calculated segregation energies ($E^{Seg}$) to identify the key chemical and geometric factors influencing solute segregation at metal/oxide interfaces, revealing the competition between these features in determining $E^{Seg}$. Moreover, the segregation behavior at a specific Fe/Y_2_O_3_ interface is predicted with high accuracy using ML models trained on data from this interface. Furthermore, it is found that the ML models could also predict solute segregation at a different Fe/Y_2_O_3_ interface with a new orientation relationship (OR), at a computational cost of less than 1/45 of that required for similar DFT calculations.

## 1. Introduction

The metal/oxide heterointerface is applied for a wide range of applications, ranging from chemically active catalysts [1] to nanoelectronics [2,3,4], biomedicine [5,6], and nuclear reactors [7,8]. These applications take advantage of the unique properties of the heterointerfaces, including optimal optical sensitivity, excellent thermal conductivity, superior electronic behavior, and high radiation resistance. The unique properties stem from the distinct atomic structure and chemistry at the interface. Both factors are affected by solute segregation to metal/oxide interfaces. Hence, an ability to predict solute segregation tendencies to metal/oxide interfaces is critical in designing novel metal/oxide composites with optimum properties.

Typically used thermodynamic-based approaches, such as Hume–Rothery rules and the Ellingham diagram, provide a simple qualitative description for the tendency of solutes to segregate to metal/oxide interface, without providing a quantitative estimation of solute segregation tendency to those interfaces. Similarly, conventional experimental and computational tools provide only a limited quantitative understanding of solute segregation behavior. For example, experimental efforts to investigate solute segregation behavior at metal/oxide interfaces in engineering alloys are complicated by the 3-D nature of the interfaces as well as interactions between multiple solutes at the interface [9,10,11]. Furthermore, these interfaces are buried deep inside the matrix, making the precise determination of atomic positions difficult. On the other hand, simulation and modeling also have their constraints. Although computational tools such as molecular dynamics (MDs) have been widely used for investigating atomic structure and chemistry of other hetero-interfaces [12,13,14], the application of MD to investigate metal/oxide interfaces is limited due to the lack of suitable empirical potentials. Another widely used method, density functional theory (DFT), an ab initio approach grounded in quantum mechanics, is limited by system size. Generally, DFT calculations can consider a maximum of a few tens to a few hundred atoms, depending on the level of approximation used [15]. Given the complex structure of the metal/oxide interfaces, DFT calculations are typically restricted to small systems, describing only fully coherent interfaces. Except for a limited number of studies, the misfit dislocation structures were not considered as such calculations often require a few hundred to a few thousand atoms [16,17,18].

In this manuscript, we utilize a hybrid approach that combines DFT calculations with machine learning (ML) for the quantitative study of solute segregation at the metal/oxide interface. ML, a rapidly advancing field within artificial intelligence (AI) and computer science, uses mathematical models and algorithms to make reliable predictions and decisions [19]. Recently, the application of ML tools is making inroads to investigate various categories of solid/solid heterointerfaces. The metal/organic heterointerface of tetracyanoethylene (TCNE) on Ag (100) was investigated using a combination of electronic structure calculations and ML models. This hybrid approach efficiently explored the potential energy surface and the formation energies of various polymorphs and defects [20]. Metal/oxide heterojunction artificial synapses for visual sensory input were studied through experiments and artificial neural networks (ANNs) [21]. Oxide/oxide heterostructures of La_1−x_Sr_x_CoO_3−_δ/La_1−x_Sr_x_MnO_3−_δ (LSCO/LSMO) were examined using first-principles simulations combined with neural networks to probe the stable structures of LSCO/LSMO bilayer interfaces across more than 50,000 compositions, enabling innovative designs for perovskite oxide-based devices [22]. Another oxide/oxide interface, LaAlO_3_/SrTiO_3_ bilayers, was simulated for convergent beam electron diffraction (CBED) patterns and investigated via a deep convolutional neural network (DCNN) to predict the step positions of a chemically diffuse interface [23]. In a separate study, high-dimensional neural networks (HDNNs) were used to develop an ML-based potential energy surface that can predict the energies of hetero-interfaces comprising 2-D graphene and 3-D Sn with reasonable accuracies [24].

This study focuses on the solute segregation behavior within the Fe/Y_2_O_3_ system, a surrogate metal/oxide interface observed in Nanostructured Ferritic Alloys (NFAs), using a combination of DFT calculations and an ML approach. NFAs are promising cladding materials for nuclear fission reactors due to their exceptional tolerance to harsh operating conditions [7,8]. We aim to comprehensively and quantitatively investigate the solute segregation behavior at Fe/Y_2_O_3_ interfaces with different orientation relationships (ORs) and dislocation conditions. Our earlier work demonstrated that misfit dislocations and ORs in Fe/Y_2_O_3_ systems can lead to variations in solute segregation behavior at different interfaces [16]. In this work, ML models are trained on DFT-calculated segregation energies ($E^{Seg}$) to identify the key chemical and geometric features that govern solute segregation behavior at a semi-coherent Fe/Y_2_O_3_ interface. The trade-off between model performance and computational cost is tested. It is found that ML models, when trained with the DFT-calculated $E^{Seg}$ of solutes at one Fe/Y_2_O_3_ interface with a particular OR, can predict the $E^{Seg}$ values of solutes at a second Fe/Y_2_O_3_ interface with a different OR. Prediction of $E^{Seg}$ at the second Fe/Y_2_O_3_ interface can be done at a fraction of the computational cost needed for similar DFT calculations without significant loss of accuracy.

## 2. Methodology

### 2.1. Electronic Structure Calculation

#### 2.1.1. Fe/Y_2_O_3_ Interface Supercells

**Figure 1:**
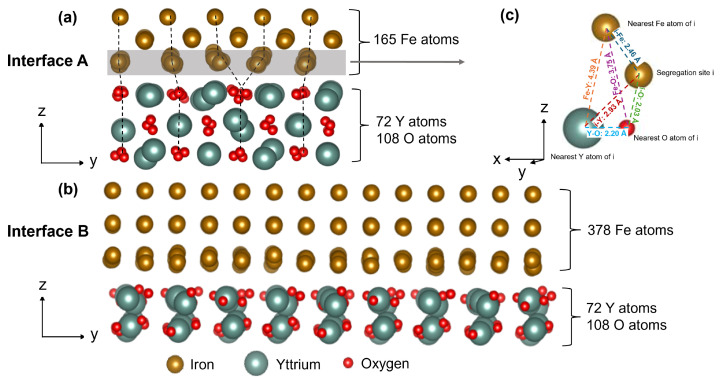
Relaxed structures of Fe/Y_2_O_3_ interface: (**a**) interface A (OR: [001]_Fe_||[100]_Y2O3_ and (010)_Fe_||(011)_Y2O3_), where the black dotted lines in the middle represent the misfit dislocation, and the gray rectangular box highlights the interfacial Fe layer; (**b**) interface B (OR: [010]_Fe_||[101]_Y2O3_ and (101)_Fe_||(010)_Y2O3_); (**c**) a visualization of geometrical surrounding a typical interfacial Fe site at interface A.

Two bi-layer supercells (interface A and B) were created for electronic structure calculations to investigate the solute segregation behavior at the Fe/Y_2_O_3_ interface in this study. Interface A (Figure 1a) consists of three layers of Fe and three layers of Y_2_O_3_ on either side of the interface. It was previously observed [17] that increasing the number of layers of metal and oxide from three to four in the case of interface A only has a minimal effect on the atomic structure and defect property at the interface. The Fe side contains 165 atoms in total, with 55 atoms in each layer, while the Y_2_O_3_ part includes 180 atoms, with a Y:O ratio of 2:3. The OR at interface A is [001]_Fe_||[100]_Y2O3_ and (010)_Fe_||(011)_Y2O3_, using the atomic structure reported in [16,17]. At interface A, a misfit dislocation is observed along the *y*-axis, as six columns of Fe atoms correspond to five columns of O atoms. A misfit dislocation is also present along the *x*-axis, where eleven columns of Fe atoms align with twelve columns of O atoms. The distance between the Fe and Y_2_O_3_ layers of interface A is determined using the interlayer distance of Fe, as reported in a previous publication [17]. In addition to interface A, interface B was created with OR, [010]_Fe_||[101]_Y2O3_ and (101)_Fe_||(010)_Y2O3_, as observed experimentally [25] (Figure 1b). At interface B, the three Fe layers consist of 378 atoms (126 atoms in each layer), while the yttria layers consist of 72 and 108 atoms of Y and O, respectively. For interface B, the distance between the Fe and Y_2_O_3_ layers was varied, and the structure with minimum energy was chosen for subsequent calculations (see Supplementary Figure S1 from the Supplementary Materials). Both interfaces A and B were optimized along the *x* and *y* directions, and the minimum energy structure was chosen for subsequent segregation energy calculations. The top and bottom layers of both oxide and metal in these two interfaces were kept fixed to mimic bulk-like conditions.

#### 2.1.2. Computational Details for DFT Calculations

All the electronic structure calculations were performed using DFT as implemented in the Vienna ab-initio Simulation Package (VASP 5.4.4) [26]. The computational parameters followed the previous research on Fe/Y_2_O_3_ interface [16,17]. The projector augmented wave (PAW) method was used, and the plane wave cut-off was 450 eV [27]. All calculations were spin polarized and the Perdew–Burke–Ernzerhof (PBE) parameterization of the generalized gradient approximation (GGA) was utilized for the exchange-correlation (XC) potential [28]. The Brillouin-zone sampling was performed using the Monkhorst–Pack scheme with a 1×1×1 K-point mesh. In all calculations, structures were relaxed until the maximum force converged to 0.05 eV/Å on each atom.

#### 2.1.3. Solute Segregation Details

In this work, the solute segregation behavior at Fe/Y_2_O_3_ interface was evaluated in terms of $E^{Seg}$ [16]:

(1) $$E^{Seg}=\left(E_{with\;solute}^{Interface}-E_{without\;solute}^{Interface}\right)-\left(E_{with\;solute}^{Bulk}-E_{without\;solute}^{Bulk}\right),$$

where $E_{with\;solute}^{Interface}$ is energy of interface with solute, $E_{without\;solute}^{Interface}$ denotes energy of interface without solute, $E_{with\;solute}^{Bulk}$ represents energy of bulk Fe with solute, and $E_{without\;solute}^{Bulk}$ is energy of bulk Fe without solute. The bulk Fe structure consists of 128 Fe atoms with a body-centered cubic structure. Here, we assume that $E^{Seg}$ does not include entropic contributions, so these are not emphasized in this study. According to Equation (1), a positive $E^{Seg}$ indicates a tendency for the solute to remain in the bulk Fe matrix, while a negative $E^{Seg}$ suggests a preference for the solute to segregate at the Y_2_O_3_ interface.

A total of 28 common substitutional solutes were considered for calculating the $E^{Seg}$ at the interfacial Fe sites for both interface A and interface B: Ag, Al, Ba, Cd, Co, Cu, Hf, Ir, K, Mg, Mo, Na, Nb, Ni, Os, Pd, Pt, Rb, Rh, Sc, Sr, Ta, Ti, V, W, Y, Zn, and Zr. To examine the effect of the dislocation, DFT calculations of $E^{Seg}$ were performed at 14 sites across interface A—five from the coherent region and nine from the misfit region. In this context, at the misfit region, two columns of Fe are shared with one column of O in Y_2_O_3_ (Figure 1a), whereas, at the coherent region, atomic columns of Fe are more aligned with atomic columns of O in Y_2_O_3_ (see Supplementary Figure S2 from the Supplementary Materials). Hence, the Fe/O ratio is higher at the misfit region compared to at the coherent region. Interfacial Fe sites were randomly selected from these coherent and misfit regions for the calculations of $E^{Seg}$. For interface B, $E^{Seg}$ calculations were performed at four randomly selected sites. In total, 28 solutes × 14 sites = 392 $E^{Seg}$ and 28 solutes × 4 sites = 112 $E^{Seg}$ were generated using DFT calculations for interface A and interface B, respectively.

### 2.2. ML Approaches

#### 2.2.1. Input Features

The parameters affecting $E^{Seg}$ at the Fe/Y_2_O_3_ interface, also known as features, are listed in Table 1. A total of 18 features were considered. These features were categorized into three groups: geometry, chemistry, and strain energy.

(1) Geometrical features describe the local environment around the segregation site, specifically, the distances between the solute site (i) and its neighboring atoms (see Figure 1c). In our previous study [16], it was observed that the solute segregation behavior at Fe/Y_2_O_3_ interfaces is influenced by the local oxygen environment. Consequently, the distance between the segregation site (i) and the nearest oxygen atom (O), denoted as i-O, is identified as a key geometrical feature. Additionally, competition may exist between the solute, the nearest Fe atom (Fe) to the solute, and the nearest Y atom (Y) to bond with the nearest O atom. Therefore, other interatomic distances—i-Fe, i-Y, Fe-Y, Fe-O, and Y-O—were also included as geometrical features that can impact $E^{Seg}$.
(2) Chemical features relate to the chemical properties of the solutes, as differences in chemical properties between the solute and Fe atoms can influence solute segregation behavior at the Fe/Y_2_O_3_ interface [16,17].
(3) Strain energy features capture how the local strain conditions influence solute segregation, expressed in terms of local strain energy [16,17]. This strain energy arises from the atomic size misfit between the solute and host Fe atom.

Similar chemical and strain energy features were considered in previous publications on the solute segregation at surfaces, grain boundaries, and interfaces. The corresponding references are presented in Table 1. The detailed definitions of the features in the chemistry and strain energy categories are provided in the Supplementary Materials.

#### 2.2.2. ML Techniques

The working principle of the ML tools used in this work is listed in this section.

##### Data Preprocessing

The compiled dataset of features contains parameters with various units and ranges, which will prevent the ML model from accurately learning the relationship between features and target, i.e., $E^{Seg}$. Therefore, data scaling is applied to reduce the bias in the units and ranges of features during model training. In this work, the data were scaled by the standardization approach.

The formula of standardization [36] is as follows:

(2) $$x_{new}=\frac{x-\mu }{\sigma },\;$$

where $x_{new}$ is the data after scaling, x is the original data, μ is mean value within the feature, and σ is standard deviation within the feature.

##### Kendall Rank Correlation

In Table 1, a total of 18 features are listed, which can potentially make the training of our ML model highly complex and computationally expensive. To overcome this issue, Kendall rank correlation analysis was applied to evaluate the correlation between each pair of features and reduce the dimensionality of the input feature space.

In Kendall rank correlation analysis [37], the correlation between two variables is assessed via the Kendall coefficient (τ), which is calculated with the following expression:

(3) $$\tau =\frac{n_{C}-n_{D}}{n(n-1)/2},\;$$

where $n_{C}$ is the number of concordant pairs, $n_{D}$ is the number of discordant pairs, and *n* is the total number of pairs.

**Figure 2:**
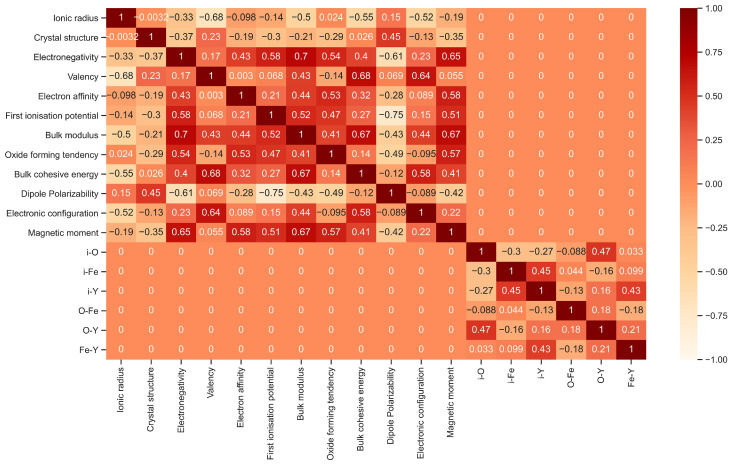
Heatmap of Kendall correlation coefficients for 18 features (data source: interface A).

τ is a numerical value that ranges between −1 and 1, which indicates a perfect negative and positive correlation, respectively. Meanwhile, 0 represents two independent variables. Therefore, if the Kendall rank correlation analysis presents a strong negative or positive correlation between two features, one of them can be eliminated, as the two features contribute similarly to the model. The results for the Kendall Rank Correlation analysis is provided in Figure 2.

##### Random Forest

Based on the dataset generated from interface A, Random Forest [38,39,40] was used to determine the relative importance of features in controlling $E^{Seg}$. Compared to other common feature importance analysis methods [41,42,43], Random Forest is particularly well-suited for identifying nonlinear relationships between features as a numerical analysis, minimizing the risk of overfitting.

**Figure 3:**
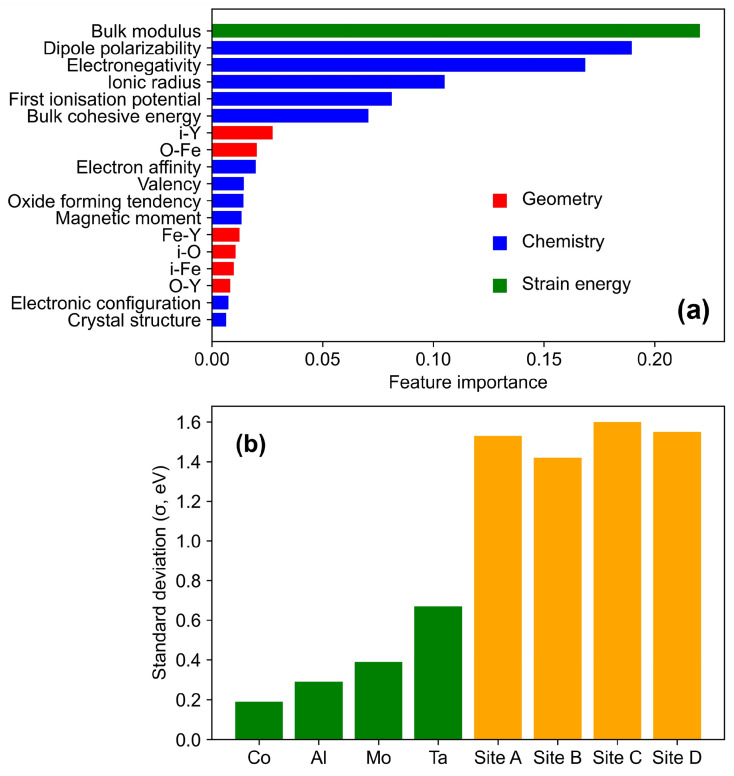
Feature importance analysis: (**a**) feature importance from Random Forest; (**b**) standard deviation (σ) of $E^{Seg}$ for the same site (orange) and for the same solute (green) (data source: interface A).

Random Forest is composed of multiple decision trees. A decision tree is a non-parametric, supervised learning technique with a tree-like structure. It involves recursively splitting the data based on decision rules at each node to improve the model’s predictive accuracy. Mean squared error (MSE) is utilized to identify the best attribute for splitting the data at the nodes of each decision tree [38,39,40]:

(4) $$MSE=\frac{1}{n}\sum_{i=1}^{n}{\left({\hat{y}}_{i}-y_{i}\right)}^{2},$$

where *n* is the total number of observations, ${\hat{y}}_{i}$ is the *i*th predicted target, and $y_{i}$ is the *i*th true target. Details of the Random Forest model are discussed elsewhere [44]. The hyperparameters of the Random Forest were tuned with the following configuration settings: 1500 trees, a maximum tree depth of 130, six features to consider at each root or decision node for the best split, one sample as the minimum at a leaf node, and two samples as the minimum required to split a root or decision node. Figure 3 presents the feature importance obtained from our Random Forest model.

##### Kernel Ridge Regression (KRR)

In this work, we used KRR as a regression model to predict $E^{Seg}$ for a given atomic site and solute. By combining ridge regression with a kernel function, KRR effectively maps the relationship between features and the numerical target into a higher-dimensional space [45]. It allows for a more detailed exploration of nonlinear relationships, making KRR a highly effective tool for numerous engineering problems [45,46,47]. Several kernel functions are feasible in KRR, such as the polynomial kernel, radial basis function (rbf) kernel, sigmoid kernel, etc. The loss function of KRR to optimize the model is expressed as follows:

(5) $$Loss=\sum_{i=1}^{n}{\left({\hat{y}}_{i}-y_{i}\right)}^{2}+\lambda \left\|w\right\|,$$

where ${\hat{y}}_{i}$ is the *i*th predicted target, $y_{i}$ is the *i*th true target, λ is a hyperparameter to describe the regularization strength, and w is the model weight vector. Details of the KRR models are presented elsewhere [44].

**Figure 4:**
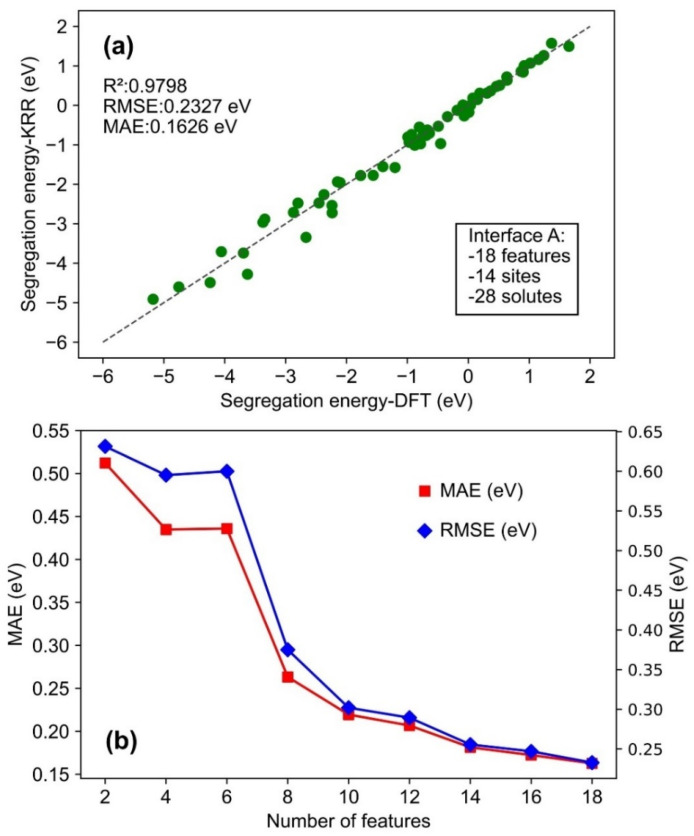
KRR model performance: (**a**) predicted $E^{Seg}$ vs. computational $E^{Seg}$ from the optimized KRR model with third-order polynomial kernel; (**b**) MAE and RMSE of KRR models with various combinations of features (data source: interface A).

**Figure 5:**
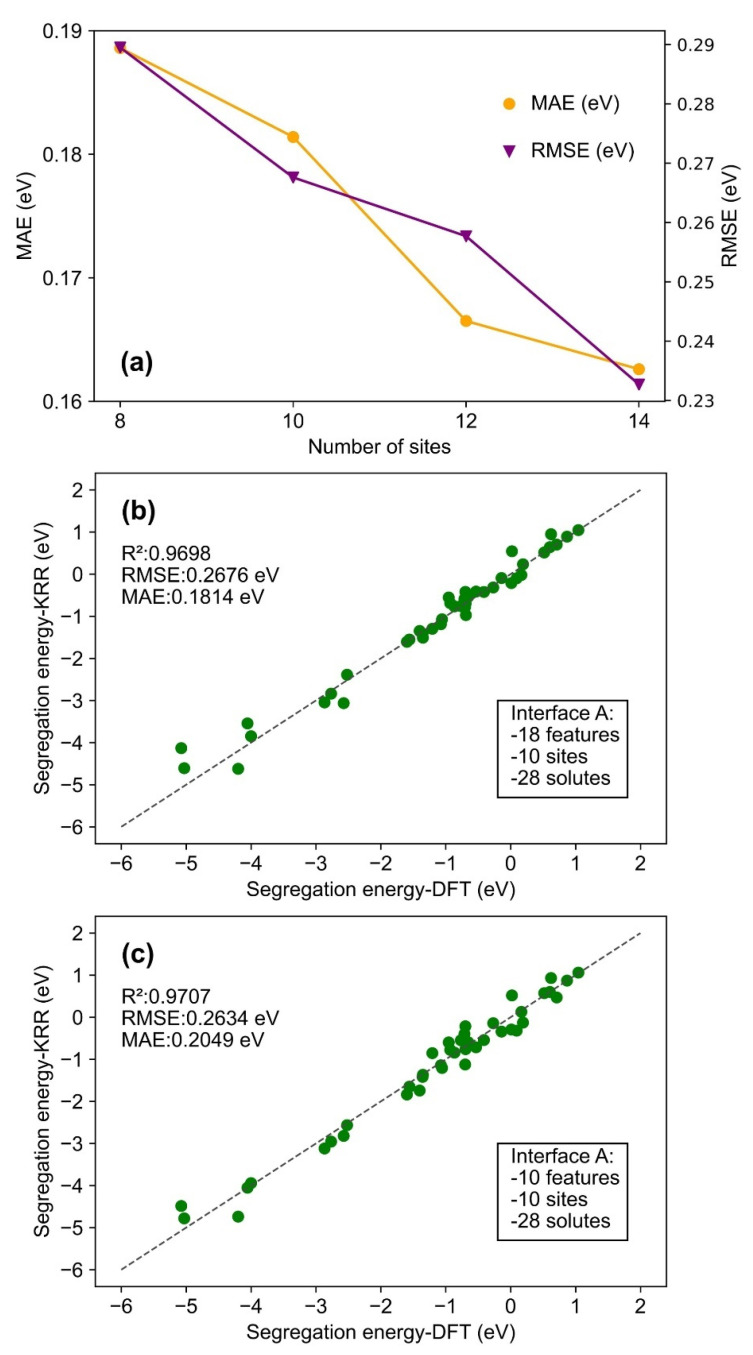
KRR model performance: (**a**) MAE and RMSE of KRR models based on 8, 10, 12, and 14 site; (**b**,**c**) predicted $E^{Seg}$ vs. computational $E^{Seg}$ from the optimized KRR model with third-order polynomial kernel with 18 and 10 features, respectively (data source: interface A).

**Figure 6:**
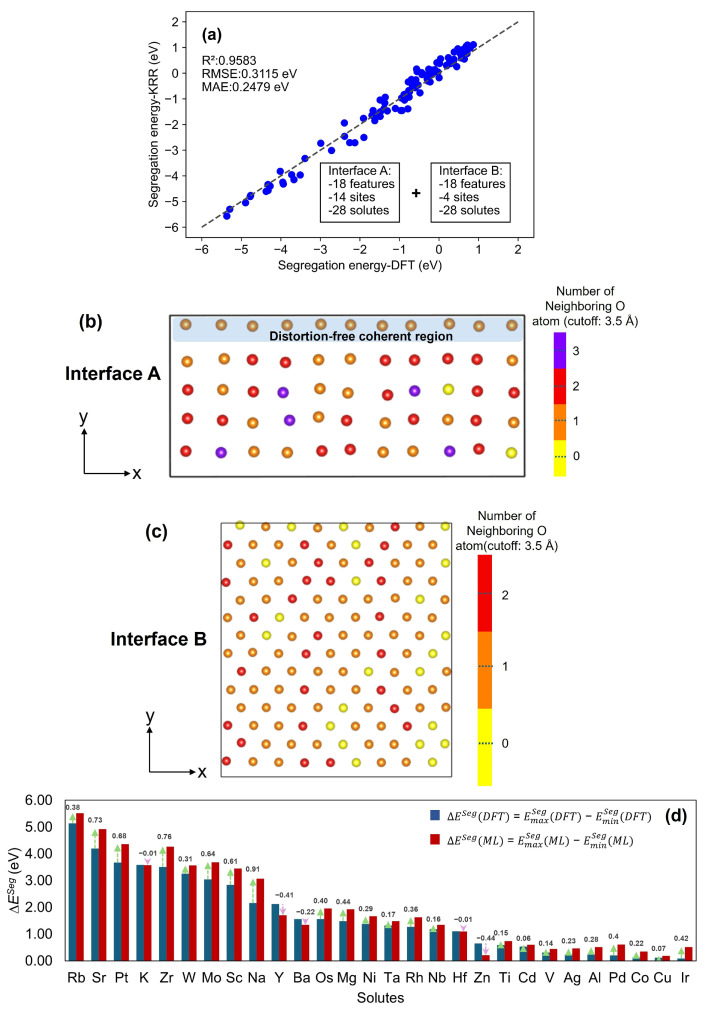
(**a**) KRR model performance of interface B: predicted $E^{Seg}$ vs. computational $E^{Seg}$ from the optimized KRR model with third-order polynomial kernel: (training set from interface A and test set from interface B); (**b**) map of number of neighboring O atoms at the interfacial Fe sites of interface A (cutoff: 3.5 Å); (**c**) map of number of neighboring O atoms at the interfacial Fe sites of interface B (cutoff: 3.5 Å); (**d**) comparison of $∆E^{Seg}$ in DFT and ML analysis (interface B), where green arrows indicate cases where $∆E^{Seg}(ML)$ exceeds $∆E^{Seg}(DFT)$, and pink arrows indicate cases where $∆E^{Seg}(ML)$ is less than $∆E^{Seg}(DFT)$.

Grid search was performed to tune the hyperparameters of the KRR models with values of hyperparameters listed in Table 2. In this table, Figure 4a shows the performance of the KRR model trained on all 18 features and 14 segregation sites (data source: interface A). Then Figure 5b presents the KRR model optimized for all 18 features and 10 segregation sites (data source: interface A). Additionally, Figure 5c illustrates the combined impact of the most important 10 features and 10 segregation sites on the KRR model performance for interface A. In Figure 6a, the KRR model trained on interface A for predicting interface B is exhibited.

In addition to KRR, Support Vector Regression (SVR) [48], Lasso regression [49], and Simple linear regression (SLR) [50] were also trained to compare the performance of various regression models. The hyperparameters employed in these models are detailed in Supplementary Table S1 of the Supplementary Materials.

##### Evaluation Metrics

The performance of each model in this study was evaluated by the coefficient of determination (R^2^) [51], mean absolute error (MAE), and root mean squared error (RMSE) [52]. The R^2^ metric is calculated based on the difference between the true values and the predicted values, as well as the difference between the true values and the average of the true values. MAE measures the average of the absolute differences between the true and predicted values. RMSE is the square root of the average squared differences between the true and predicted values, assessing the variance of the residuals. The formula for each evaluation metric is listed as follows:

(6) $$R^{2}=1-\frac{\sum_{i=1}^{n}{\left(y_{i}-{\hat{y}}_{i}\right)}^{2}}{\sum_{i=1}^{n}{\left(y_{i}-{\bar{y}}_{i}\right)}^{2}},$$

(7) $$MAE=\frac{1}{n}\sum_{i=1}^{n}\left|{\hat{y}}_{i}-y_{i}\right|,$$

(8) $$RMSE=\sqrt{\frac{1}{n}\sum_{i=1}^{n}{\left({\hat{y}}_{i}-y_{i}\right)}^{2}},$$

where *n* is total number of observations, ${\bar{y}}_{i}$ is the mean value of the target, ${\hat{y}}_{i}$ is the predicted target, and $y_{i}$ is the true target. To score the model performance in this work, R^2^, MAE, and RMSE were calculated using DFT calculated $E^{Seg}$ ${(y}_{i})$ and ML predicted $E^{Seg}$ ${(\hat{y}}_{i}$).

All the ML techniques used in this manuscript were implemented in the scikit-learn package, version 0.24.1.

## 3. Results and Discussion

### 3.1. Feature Importance Analysis

#### 3.1.1. Heatmap Collected from Kendall Rank Correlation

Figure 2 presents the results of the Kendall rank correlation coefficient analysis in the form of a heatmap for all 18 features. The features are listed along both the vertical and horizontal axes. The values in the heatmap indicate the Kendall correlation coefficient (τ) between the features corresponding to the vertical and horizontal directions. In Figure 2, geometry–chemistry feature pairs and geometry–strain energy feature pairs show no correlation (τ = 0), as expected intuitively. The geometry group of features is derived from the local structure of the segregation site, including the distances between the segregation site and neighboring atoms. The chemistry group of features consists of features related to the solute’s chemical properties. As such, the geometry features are based on the atomic structure of interface A, which is independent of the solute type.

Except for the geometry-related feature pairs, all chemistry–strain energy feature pairs show a nonzero correlation, as chemistry and the bulk modulus of the solute are related. In Figure 2, τ between the bulk modulus and electronegativity is 0.7, which is the strongest correlation among the feature pairs. This relationship arises because the bulk modulus is a measure of a material’s compressibility: a higher bulk modulus indicates that the material is less compressible, while a lower bulk modulus suggests that the material is more easily compressed [53]. The compressibility of materials is linked to the electron density of atoms, as higher electron densities typically result in lower compressibility [54]. Then, a higher electronegativity, which measures an atom’s ability to attract and retain electrons, is associated with a higher electron density in atoms [54]. Therefore, solutes with higher electronegativity tend to have higher electron densities and, consequently, a higher bulk modulus. This explains the positive correlation (τ = 0.70) between bulk modulus and electronegativity observed in Figure 2. The correlation analysis indicates that there are no feature pairs with a very strong correlation (τ > 0.9 or <−0.9) [55], meaning that none of the features will be eliminated based on this criterion. As a result, all features will be retained, since each of them contributes uniquely toward $E^{Seg}$.

#### 3.1.2. Numerical Feature Importance Generated from Random Forest

The combined effect of all the features on $E^{Seg}$ was evaluated using a Random Forest model based on the $E^{Seg}$ dataset generated for interface A. The results of the feature importance analysis are shown in Figure 3a, where the feature importance is displayed numerically along the horizontal axis, and the features are listed along the vertical axis.

In Figure 3a, the features are ranked according to their importance, from high to low, illustrating the relative contributions of geometry, chemistry, and strain energy in affecting $E^{Seg}$. In this figure, a higher feature importance value indicates a stronger influence of the feature in determining $E^{Seg}$ [38]. The top six most important features all belong to the chemistry and strain energy groups, demonstrating that these factors play a more dominant role than geometry features in predicting $E^{Seg}$. To further understand the relative role of chemistry/strain energy-related features and geometry-related features, the standard deviation (σ) in $E^{Seg}$ for the 28 solutes at a particular site, as well as the σ in $E^{Seg}$ for each solute across 14 sites, were both calculated. The σ values of four solutes and four segregation sites are randomly chosen and presented in Figure 3b. In this figure, the σ of $E^{Seg}$ among solutes, when the geometry-related features are fixed (i.e., 28 solutes at the same site), is much larger than the σ of $E^{Seg}$ among sites when chemistry and strain energy-related features are held constant (i.e., the same solute across 14 sites). This indicates that $E^{Seg}$ is more sensitive to the chemistry of the solute and the local strain energy than to the local geometry. This implies that the tendency of solutes to segregate from bulk Fe to the metal/oxide interface is higher compared to the in-plane variation in segregation tendency among different Fe sites, which was effectively captured by our ML model.

From Figure 3a, the most important feature is the bulk modulus, a feature needed to determine strain energy [29]. During solute segregation at the Fe/Y_2_O_3_ interface, a substitutional solute causes local spatial distortion due to the atomic size misfit between the solute and the host Fe atoms, which may hinder solute segregation [56]. The most important feature from the chemistry group is dipole polarizability, which defines the solute’s ability to form dipoles in a constant electric field. Polarizable atoms are typically more flexible and tend to have a weaker pull on their electrons towards the nucleus [57]. At metal/oxide interfaces, such as Fe/Y_2_O_3_, strong electrostatic fields arise from the mismatch in electronic structure and charge distribution between Fe and Y_2_O_3_ phases. Solutes with higher dipole polarizability can more readily distort their electron clouds in response to these fields, resulting in a lower energy at the interface. This effect leads to a more negative $E^{Seg}$, promoting solute segregation to the interface. Additionally, polarizable solutes are less electronegative and are more likely to bond with highly electronegative atoms, enhancing the solute’s tendency to segregate at the Fe/Y_2_O_3_ interface. This observation is also supported by the correlation between dipole polarizability and electronegativity presented in Figure 2 (τ = −0.61). Among the geometry-related features, the distance between the solute and the nearest Y atom (i-Y) is the most significant, highlighting the competition between the solute and its nearest Y atom to chemically interact with neighboring O atom(s).

### 3.2. ML Models for Predicting $E^{Seg}$

#### 3.2.1. Interface A: Role of Number of Features in Predicting $E^{Seg}$ Using KRR Model

KRR was applied to the solute segregation data for interface A. The dataset was randomly split into training and test sets with a ratio of 85:15. Figure 4a exhibits the performance of the KRR model trained using all 18 features. In this figure, the test data are presented in a parity plot, where the *x*-axis (i.e., horizontal axis) represents the DFT-calculated $E^{Seg}$ values, and the *y*-axis (i.e., vertical axis) denotes the KRR-predicted $E^{Seg}$. In this regard, the previous DFT-calculated solute segregation tendency was confirmed by experiments. For example, the non-uniform (between the misfit and coherent region) segregation tendency of solutes like Cr at metal/oxide interfaces predicted based on DFT-calculated $E^{Seg}$ values was previously verified experimentally [16]. In Figure 4a, the R^2^ value of 0.9798 (close to 1) indicates good agreement between ML-predicted and DFT calculated values of $E^{Seg}$ [51]. Furthermore, all the data fall close to the parity line, verifying that the KRR model can predict the $E^{Seg}$ of various solutes at the Fe/Y_2_O_3_ interface with high fidelity.

It can be observed that, for test data with highly negative $E^{Seg}$ values, ranging from −5 to −2 eV (in Figure 4a), the data points are slightly scattered away from the parity line. It is observed that all these data correspond to solutes with large ionic sizes, such as Ba (2.53 Å), K (2.43 Å), Rb (2.65 Å), etc., compared to Fe (1.56 Å). The strain energy arising from the atomic misfit is represented by the bulk modulus in our study. The sole strain energy feature, i.e., bulk modulus, may not fully capture all the local strain energy contribution at the Fe/Y_2_O_3_ interface, leading to a higher prediction error for the $E^{Seg}$ of large-sized solutes. This is unlike previous studies on solute segregation at grain boundaries, where bulk modulus was used to describe the strain energy due to atomic misfit [56].

Besides the KRR, SVR, Lasso regression, and SLR were also employed to train models for predicting $E^{Seg}$. The hyperparameters, R^2^, RMSE, and MAE of these models are provided in Supplementary Table S1. Among all the models, the performance of the KRR model stands out with the best evaluation scores.

Additionally, several other KRR models were trained to examine the impact of the number of features on model performance. Comparisons of MAE and RMSE against the number of features for each model are shown in Figure 4b. Nine machine learning models were trained using different numbers of input features (2, 4, 6, 8, 10, 12, 14, 16, and 18). For models utilizing fewer than 18 features, only features with the highest importance in Figure 3a were selected. This feature selection ensured that the most informative variables were prioritized when constructing models with reduced feature sets. Further details regarding the performance of each model can be found in Supplementary Table S2 within the Supplementary Materials. It is found that both MAE and RMSE follow a similar upward trend as the number of features decreases from 18 to 2 in Figure 4b. This trend indicates that a larger number of features can better capture the relationship between the features and the segregation behavior at the Fe/Y_2_O_3_ interface, thereby enhancing the performance of the ML model with lesser prediction errors for $E^{Seg}$. In Figure 4b, both MAE and RMSE show significant changes in error as the number of features increases from two to eight, but, after that, the decline becomes smoother. It can be concluded that a feature size between 8 and 12 strikes a good balance between model performance and complexity for the KRR model.

#### 3.2.2. Interface A: Role of the Data Size in Predicting $E^{Seg}$ Using the KRR Model

The next step is to investigate the impact of sample size on the performance of ML models. While the models discussed so far are based on data calculated from 14 sites at interface A, in this section, we explore the role of the number of sites in the dataset and its effect on the accuracy of ML-predicted $E^{Seg}$. In this regard, the number of segregation sites in the datasets was decreased incrementally from 14 to 8 sites (in a step of 2) by randomly selecting the sites equally between the coherent and misfit regions, while keeping the number of solutes fixed at 28. The performance of these KRR models is presented in Figure 5a. The detailed performance metrics for these models can be found in Supplementary Table S3 within the Supplementary Materials. As the number of sites considered is decreased from 14 (392 $E^{Seg})$ to 10 (280 $E^{Seg}$), RMSE and MAE are both increased by 15.00% (from 0.2327 eV to 0.2676 eV) and 11.56% (from 0.1626 eV to 0.1814 eV), respectively.

The KRR model optimized for 10 sites is shown in Figure 5b. In this figure, the test data lie close to the parity line. The R^2^ of this model is 0.9698, indicating that the model explains 96.98% of the variance in the test data with relatively small errors. Despite the increase in error from 14 sites to 10 sites, the simplified sample size leads to a reduction by 28.20% in computational cost of DFT, from 3365 h to 2416 h (the total CPU time used). This trade-off is important when balancing performance with efficiency. The interface A, with 345 atoms, is a relatively large supercell, implying that reduced sample size helps keep the computational cost manageable without drastically compromising the model’s performance.

In Figure 5c, the combined effect of the number of features and number of sites on the performance of the KRR model is demonstrated using data from the 10 most important features (from Random Forest) and 10 interfacial sites. In this analysis, the performance of the KRR model trained with a reduced feature size and sample size is compared with two other models: the KRR model in Figure 5b, and the KRR model in Figure 4a (see Table 3). The R^2^ and RMSE for the model in Figure 5c are very similar to those in Figure 5b, while the MAE for the model in Figure 5c is increased by 12.95%. Feature size is reduced by 44.44%, and sample size is reduced by 28.57% (between Figure 4a and Figure 5c). Despite these reductions, the increase in RMSE is only 13.19%, and the MAE only increases by 26.01%. These increases in error are relatively modest, indicating that the model still performs well despite the significant reductions in both feature and sample sizes. Hence, the combined reduction in feature size and sample size (from 18 features and 14 sites to 10 features and 10 sites) allows for significant DFT computational cost savings and simplification of the trained model. The increase in error metrics (RMSE and MAE) is small relative to the size reductions, demonstrating that this trade-off can still yield strong model performance while balancing model complexity and computational efficiency. This reduction is particularly useful for studying complex systems like the Fe/Y_2_O_3_ interface, where maintaining high accuracy while reducing computational demand is crucial.

#### 3.2.3. Information Transfer Between Interface A and B

To predict $E^{Seg}$ at interface B, the KRR model was trained using the full dataset of 392 segregation data obtained for interface A. Results of the test data are presented in Figure 6a. All the test data for interface B are located along the parity line without outliers in the parity plot, suggesting excellent model accuracy with no significant outliers. Figure 6a indicates excellent transferability of $E^{Seg}$ between interface A and interface B. This can also be observed from the high R^2^ (0.9583), reasonable RMSE (0.3115 eV), and MAE (0.2479 eV) of the KRR model. In addition, to evaluate the overall performance of the KRR model for all solute segregation at interface B, the RMSE and MAE for the predicted $E^{Seg}$ of each individual solute at interface B were calculated and analyzed (see Supplementary Table S4). When normalized by the range of calculated $E^{Seg}$ values (6.2340 eV), the resulting error rates based on RMSE fall between 1.88% and 8.06% (with an average of 4.20%), and those based on MAE lie between 1.67% and 7.80% (with an average of 3.59%). These low errors of individual solutes further highlight the model’s strong predictive performance. Thus, there is a high possibility that the ML model can effectively predict $E^{Seg}$ in a metal/oxide interface system with different characteristics (larger supercell size and/or different OR). In particulate composite materials, particles embedded in the matrix often exhibit multiple facets, with each facet having a different OR with the matrix. Our results indicate that it is not necessary to calculate the $E^{Seg}$ for each facet individually. Instead, once $E^{Seg}$ is calculated for one facet, ML can be applied to predict $E^{Seg}$ for the other facets, thus paving a significant reduction in computational cost to investigate segregation tendency at the oxide-particle/matrix interface.

At interface A, a misfit dislocation is observed (Figure 1a), whereas, at interface B, a twist component exists, presenting a challenge in understanding the dislocation architecture at this interface. Figure 6b,c presents a map of the number of neighboring O atoms at the interfacial Fe sites of interface A and interface B, respectively, using a cutoff distance of 3.5 Å. From Figure 6b, it is observed that each Fe atom is bonded to one O atom in the distortion-free coherent region, whereas the remaining interfacial Fe atoms have zero, one, two, or three O atoms as neighbors at interface A. Fe and O atoms within the distorted region experience significant lattice distortion due to interfacial strain. As a result, several interfacial Fe sites in this region have more than one O neighbor within the cutoff, whereas, in the distortion-free region, each interfacial Fe site is coordinated with only one nearest O neighbor within the cutoff at interface A. Similarly, interface B also exhibits a non-uniform distribution of O atoms (0–2 neighboring O atoms) for each interfacial Fe atom, providing evidence that interface B is not fully coherent (see Figure 6c). This finding indicates that the combined DFT calculation and ML approach can predict the solute segregation pattern of a metal/oxide interface with different OR, where explicit dislocation features are not present.

Although it has been observed that chemistry and strain energy-related features play a dominant role in predicting $E^{Seg}$, the effect of local geometry on solute segregation behavior was also further studied. That is, we examined the ability of the model to predict variations in $E^{Seg}$ within the interfacial plane that arise from the misfit dislocation structure of the interface. $∆E^{Seg}$, which indicates the range ($E_{max}^{Seg}-E_{min}^{Seg}$) of $E^{Seg}$ values across the four segregation sites for each solute, was calculated for interface B, and the results are presented in Figure 6d. In this figure, the solutes are organized according to their $∆E^{Seg}$ values from DFT (indicated by the blue bars), along with the ML predicted $∆E^{Seg}$ (indicated by the red bars). The figure also displays the error values in predicting $∆E^{Seg}$, which is calculated as $∆E^{Seg}(ML)-∆E^{Seg}(DFT)$. Solutes with large $∆E^{Seg}$ differences (>0.5 eV) tend to have bigger atomic size than Fe, which includes Sr, Pt, Zr, Mo, Sc, and Na, all of which are positioned at the higher $∆E^{Seg}(DFT)$ in Figure 6d. The average $∆E^{Seg}$ difference as a fraction of the average $∆E^{Seg}(DFT)$ is 22.22% for these six solutes. While the magnitudes of $∆E^{Seg}$ from DFT calculations and ML do not perfectly match, ML captures the overall trend in $∆E^{Seg}$ among the four different sites in interface B. Even though chemistry and strain energy dominate the description of the solute segregation behavior at the Fe/Y_2_O_3_ interface, the local geometry of the interface plays a vital role in the in-plane variation in $E^{Seg}$ at the interfacial Fe sites. It can be concluded that the ML model is capable of describing the influence of local geometry on solute segregation behavior at a metal/oxide interface.

**Figure 7:**
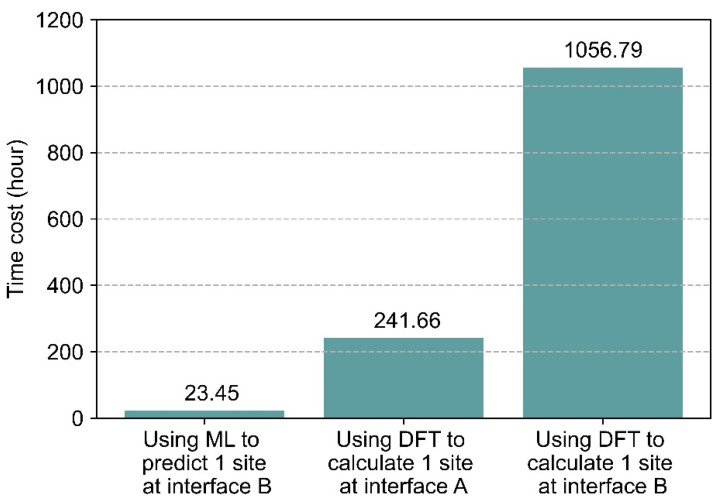
Comparison of computational cost for ML to predict $E^{Seg}$ at one site in interface B with computational cost to calculate $E^{Seg}$ at one site in interface A and B using DFT calculations.

Along with developing a quantitative model for predicting solute segregation behavior at the Fe/Y_2_O_3_ interface system, ML also provides significant potential for reducing the computational cost of studying these interfaces. With the help of ML, more extensive analyses can be conducted of interfaces, enabling investigations that would have been otherwise challenging, if not impossible, with traditional DFT calculations. Figure 7 compares the total CPU time required for ML predictions of $E^{Seg}$ at interface B, along with the time required for DFT calculations of $E^{Seg}$ at both interface A and interface B. The time required to apply ML for predicting the segregation of 28 solutes at a single site on interface B was 23.45 h, whereas calculating the same 28 solute segregations for the same interface using DFT requires 1056.79 h. At interface A, it took 241.66 h to calculate the segregation of 28 solutes at a single site. It can be observed in Figure 7 that, as the number of atoms increases by 1.62 times from interface A to B (345 vs. 578), the DFT computational time grows by a factor of 4.37, as expected (as the system size increases, the computational time required for DFT calculations grows as O(*N*^3^) where *N* = number of electrons in the supercell). However, DFT-based calculation of $E^{Seg}$ of 28 solutes of interface B requires more than 45 times compared to the ML-based approach. It is found that combining ML with DFT calculations can dramatically improve our ability to investigate solute segregation behavior at metal/oxide interface, especially for large interfaces with complex structures and dislocation conditions. Furthermore, ML enables information transfer across different interfaces, providing a more systematic and efficient framework for research and facilitating the exploration of a broader range of metal/oxide interfaces.

## 4. Conclusions

In this study, a combination of DFT calculations and an ML hybrid approach was used to explore the solute segregation behavior at the Fe/Y_2_O_3_ interface in terms of $E^{Seg}$. The feature importance analysis demonstrated that chemistry and strain energy-related features have a greater influence on $E^{Seg}$ prediction compared to geometry-related features. Additionally, the ML model was used to accurately predict $E^{Seg}$ at interface A. Later, a balance among model performance, complexity, and computational cost was achieved by using data of 10 features and 10 segregation sites only. Furthermore, ML trained on $E^{Seg}$ at interface A was used to successfully predict $E^{Seg}$ at interface B, requiring less than 1/45 of the computational cost of similar DFT calculations. This work highlights a new perspective on the study of solute segregation behavior at the metal/oxide interface: by utilizing a subset of computational data from a single metal/oxide interface, ML can effectively guide research across a broad range of metal/oxide interfaces in a more time- and cost-efficient manner.

While the combined use of DFT calculations and an ML approach accelerates the study of solute segregation behavior at the Fe/Y_2_O_3_ interface, limitations remain.

The local strain condition at the segregation site was only described by the bulk modulus of the solute, similar to the results previously reported in the literature, representing the elastic strain energy of solute segregation at the grain boundary. However, the Fe/Y_2_O_3_ interface is more complex than the grain boundary, creating a gap between the computational and predicted $E^{Seg}$.

Additionally, in this study, the segregation tendency of individual solute atoms was examined. But real alloy systems may involve a multi-solute environment at Fe/Y_2_O_3_ interfaces. To adequately predict the solute segregation tendency for a multi-solute environment, the current model can be extended by adding additional features describing solute–solute interactions.

## Figures and Tables

**Table 1:** Input features for ML.

Geometry	Chemistry	Strain Energy
i ^(a)^-Fe ^(b)^ (Å)	Ionic radius (Å) [16]	Bulk modulus (GPa) [29]
i ^(a)^-Y ^(c)^ (Å)	Crystal structure [16]	
i ^(a)^-O ^(d)^ (Å)	Valency [16]	
Fe ^(b)^-O ^(d)^ (Å)	Electronegativity [16]	
Fe ^(b)^-Y ^(c)^ (Å)	Oxide forming tendency (eV/atom) [16]	
Y ^(c)^-O ^(d)^ (Å)	Bulk cohesive energy (eV/atom) [30]	
	Electron affinity (eV/atom) [31]	
	First Ionization potential (eV/atom) [32]	
	Dipole polarizability (a.u.) [33]	
	Electronic configuration [34]	
	Magnetic moment (BM) [35]	

^(a)^ segregation site; ^(b)^ nearest Fe atom of segregation site; ^(c)^ nearest Y atom of segregation site; ^(d)^ nearest O atom of segregation site.

**Table 2:** Hyperparameters used in KRR models.

KRR Model	Alpha ^(a)^	Gamma ^(b)^	Degree ^(c)^	Kernel ^(d)^
Figure 4a	0.8	0.1	3	polynomial
Figure 5b	0.4			
Figure 5c	0.1			
Figure 6a	1			

^(a)^ regularization strength; ^(b)^ coefficient of the polynomial kernel function; ^(c)^ degree of the polynomial kernel function; ^(d)^ mathematical functions used in the KRR model.

**Table 3:** Comparisons of model performance for various numbers of sites and features.

KRR Model	Number of Sites	Number of Features	R^2^	RMSE (eV)	MAE (eV)
Figure 4a	14	18	0.9798	0.2327	0.1626
Figure 5b	10	18	0.9698	0.2676	0.1814
Figure 5c	10	10	0.9707	0.2634	0.2049

## Data Availability

The original contributions presented in this study are included in the article/Supplementary Materials. Further inquiries can be directed to the corresponding author(s).

## Supplementary Materials

The following supporting information can be downloaded at: https://www.mdpi.com/article/10.3390/molecules30163344/s1, Figure S1: Energy of interface B with varying distances along z-direction between Fe and Y_2_O_3_ layers; Figure S2: Coherent and misfit regions identified at the interface A (interfacial Fe sites were randomly selected from these coherent and misfit regions for the calculation of $E^{Seg}$); Table S1: Optimized hyperparameters and performance of regression models; Table S2: Optimized KRR model performance for datasets with various features; Table S3: Optimized KRR model performance for datasets with various dataset sizes; Table S4: Optimized KRR model performance for different solutes.
