# Supplementary material for: Prediction of Solute Segregation at Metal/Oxide Interfaces Using Machine Learning Approaches
Source: Molecules. 2025 Aug 11;30(16):3344. doi: 10.3390/molecules30163344 (PMC12388548; doi:10.3390/molecules30163344)
Supplement: Supplementary file 1 [file molecules-30-03344-s001.zip › molecules-3648369-supplementary.pdf]

Supplementary Information

Prediction of Solute Segregation at Metal/Oxide Interfaces Using Machine Learning Approaches

Yizhou Lu[1], Blas Pedro Uberuaga[2], Samrat Choudhury[1,*]
[1]Department of Mechanical Engineering, University of Mississippi, University, MS 38677, USA
[2] Materials Science and Technology Division, Los Alamos National Laboratory, Los Alamos, NM 87545, USA

[*] **Corresponding author**

Definitions of features in Table 1

1. Ionic radius (Å): the distance from the nucleus of an ion up to which it has an influence on its electron cloud.
2. Crystal structure: the specific and repeating pattern of atoms (molecules or ions) within a crystal.
3. Valency: the ability to be combined with other atoms to form chemical compounds or molecules.
4. Electronegativity: the tendency of an atom to attract electrons toward itself.
5. Oxide forming tendency (eV/atom): the Ellingham diagram plots the Gibbs free energy change (ΔG) for each oxidation reaction as a function of temperature. The lower the line on the Ellingham diagram, the more thermodynamically stable the oxide is compared to other oxides.
6. Bulk cohesive energy (eV/atom): the energy released when atoms arrange themselves in a crystalline structure, compared to when they are in the gaseous state.
7. Electron affinity (eV/atom): the energy released when an electron is added to a neutral atom to form a negatively charged ion.

8. First ionization potential (eV/atom): the energy needed to remove a valence electron from a neutral atom.
9. Dipole polarizability (a.u.): a measure of the response of a system of charged particles to an electric field. At metal-oxide interfaces such as Fe/Y_2O_3, strong electrostatic fields arise from the mismatch in electronic structure and charge distribution between Fe and Y_2O_3 phases. Solutes with higher dipole polarizability can more readily distort their electron clouds in response to these fields, resulting in a lower energy at the interface. This effect leads to a more negative E^{Seg}, promoting solute segregation to the interface rather than immobilization in the bulk Fe.
10. Electronic configuration: the distribution of electrons in its atomic orbitals.
11. Magnetic moment (BM): the material's tendency to align with a magnetic field. In ferromagnetic hosts such as Fe, solutes with magnetic moments that align or interact favorably with the host's magnetic moment can be energetically stabilized at the interface, resulting in a more negative E^{Seg} and thereby enhancing the tendency of the solute to be trapped at the interface.
12. Bulk modulus (GPa): The local strain energy at heterointerfaces is inherently complex due to variations in atomic structure, bonding environments, and lattice mismatches. Currently, there is no published mathematical expression that accurately captures the local strain energy. Thus, we surveyed the literature on grain boundary segregation, where the local elastic strain energy (E_{el}) can be described using the Langmuir-McLean theory, as shown below [S1]:

$$E_{el} = \frac{24\pi K G r_0 (r_1 - r_0)^2}{3K + 4G}, \qquad (S1)$$

where K is the solute's bulk modulus, G is the matrix shear modulus, r_0 and r_1 are the atomic radii of the matrix and solute, respectively.

In this study, G (the matrix shear modulus) and r_0 (the matrix atomic radius) are intrinsic properties of the Fe phase and remain constant across all solute segregation cases. In ML, such features are referred to as constant features. Since they do not vary across the dataset, constant features do not contribute to differentiating between data points and can be safely removed without affecting the model's capability to learn patterns among the datasets. For the atomic radius of the solute, it generally decreases due to increasing nuclear charge across a period in the periodic table (left to right). We have already used ionic radius as one of the chemical features, and the ionic radius exhibits a similar trend but is also strongly influenced by the ion's charge state. As a result, the effects of atomic and ionic radii partially overlap in their influence on E^{Seg}. To avoid redundancy, we ultimately included only K (the solute's bulk modulus) as a descriptor for local strain energy.

Optimization of interface B along *z*-direction

To optimize interface B along the *z*-direction, the interlayer distance between the Fe slab and the Y_2O_3 slab was varied from 2.25 to 2.57 Å. The total energy of each resulting structure was calculated and compared, as shown in Supp. Figure 1. The minimum energy was found at a separation of 2.41 Å, which was then used in all subsequent calculations.

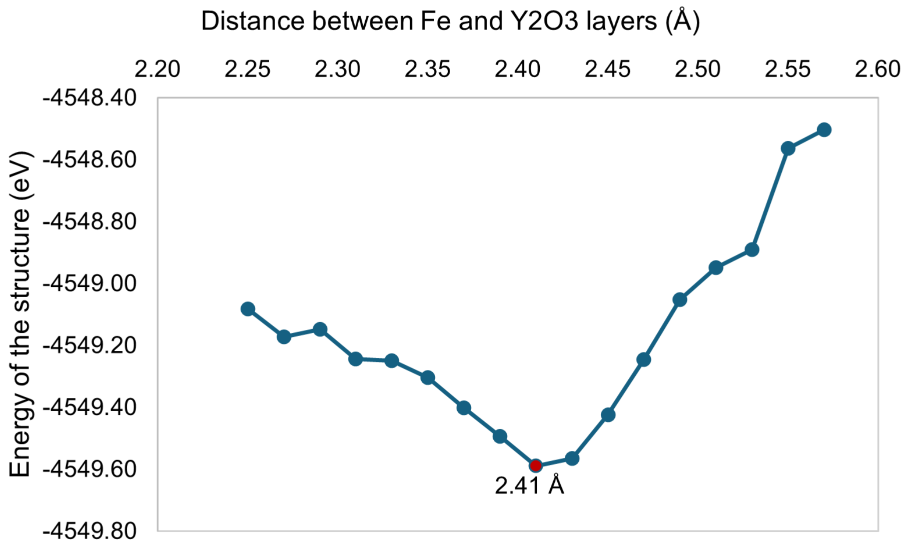

Figure S1. Energy of interface B with varying distances along *z*-direction between Fe and Y_2O_3 layers.

The definition of coherent region and misfit region

At the misfit region two columns of Fe are shared with one column of O in Y_2O_3, whereas at the coherent region atomic columns of Fe are more aligned with atomic columns of O in Y_2O_3 as presented below.

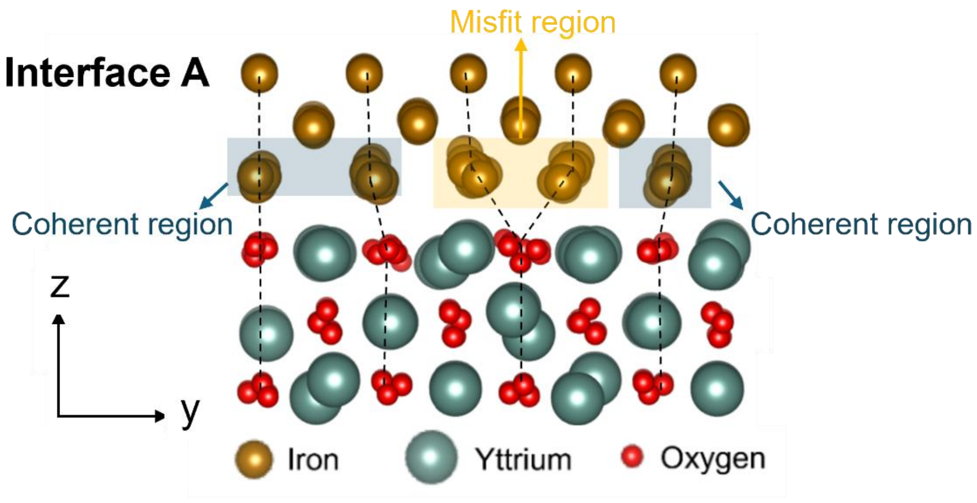

Figure S2. Coherent and misfit regions identified at the interface A (interfacial Fe sites were randomly selected from these coherent and misfit regions for the calculation of E^{Seg}).

The tuned hyperparameters and performance of regression models

KRR, SVR, Lasso regression, and SLR were used to train models for predicting E^{Seg} (data source: interface A). The hyperparameters, R^2, RMSE, and MAE of these models are provided in the table below. Among all the models, the KRR model demonstrates the strongest performance, achieving the highest evaluation scores.

Table S1. Optimized hyperparameters and performance of regression models.

Regression	Hyperparameters	R^2 (eV)	RMSE (eV)	MAE (eV)
KRR	alpha[a)] = 0.8<br>gamma[b)] = 0.1<br>degree[c)] = 3<br>kernel[d)] = 'poly'	0.9798	0.2327	0.1627
SVR	C[e)] = 0.9<br>gamma[b)] = 0.1<br>epsilon[f)] = 0.1<br>degree[c)] = 3<br>kernel[d)] = 'poly'	0.9694	0.2867	0.2057
Lasso	alpha[a)] = 0.1	0.8775	0.5746	0.4766
SLR	None	0.9016	0.5152	0.4119

[a)] regularization strength; [b)] coefficient of the polynomial kernel function; [c)] degree of the polynomial kernel function; [d)] mathematical functions used in model; [e)] regularization parameter; [f)] width of the margin.

The effect of the number of features on model performance

Numerical RMSE and MAE for models with different number of features are listed in the table below (data source: interface A). For models with fewer than 18 features, only the top-ranked features, as determined by their importance in Figure 3(a), were selected.

Table S2. Optimized KRR model performance for datasets with various features.

Number of features	RMSE (eV)	MAE (eV)
2	0.6314	0.5122
4	0.5952	0.4348
6	0.5978	0.4359
8	0.3211	0.2365
10	0.3019	0.2194

12	0.2893	0.2067
14	0.2555	0.1813
16	0.2019	0.1473
18	0.2327	0.1626

The effect of sample size on model performance

Numerical RMSE and MAE of the KRR models with different number of segregation sites are presented in the table below (data source: interface A). The number of segregation sites were randomly selected, and they were equally divided between the coherent and misfit regions.

Table S3. Optimized KRR model performance for datasets with various dataset sizes.

Number of Sites	RMSE (eV)	MAE (eV)
8	0.2895	0.1886
10	0.2676	0.1814
12	0.2577	0.1665
14	0.2327	0.1626

An analysis of model performance across individual solutes

The RMSE and MAE for the predicted E^{Seg} of each individual solute at interface B were presented in the table below.

Table S4. Optimized KRR model performance for different solutes.

Solute	RMSE (eV)	MAE (eV)
Ag	0.1736	0.1517
Al	0.2700	0.2158
Ba	0.2527	0.2145
Cd	0.1331	0.1100
Co	0.1965	0.1762
Cu	0.1374	0.1285
Hf	0.3283	0.2913
Ir	0.3951	0.3567
K	0.1611	0.1592
Mg	0.3399	0.2678
Mo	0.2475	0.1882
Na	0.5026	0.4864

Nb	0.1926	0.1783
Ni	0.2558	0.2247
Os	0.3045	0.2334
Pd	0.1670	0.1222
Pt	0.3376	0.3016
Rb	0.1353	0.1043
Rh	0.2122	0.1604
Sc	0.4307	0.4057
Sr	0.4363	0.3187
Ta	0.2523	0.2421
Ti	0.1172	0.1054
V	0.3035	0.2686
W	0.1822	0.1571
Y	0.3538	0.3061
Zn	0.2307	0.1484
Zr	0.2819	0.2347

References:

[S1] McLean, D. *Grain Boundaries in Metals*; Clarendon Press: Oxford, UK, **1957.**
